# Attention mechanisms underlying dual‐color digital visual search based on Schulte grid: An event‐related potential study

**DOI:** 10.1002/brb3.2471

**Published:** 2022-01-08

**Authors:** Aiqiang Lu, Dongmei Wang, Shengxi He, Qiuyi Zhongcheng, Wei Zhang, Zezhi Li

**Affiliations:** ^1^ College of Psychology Shenzhen University Shenzhen Guangdong China; ^2^ Shenzhen Health Development Research Center Shenzhen Guangdong China; ^3^ Institute of Psychology CAS and Department of Psychology University of Chinese Academy of Sciences Beijing China; ^4^ Department of Psychiatry The Affiliated Brain Hospital of Guangzhou Medical University Guangzhou China; ^5^ Guangdong Engineering Technology Research Center for Translational Medicine of Mental Disorders Guangzhou China

**Keywords:** attention, color priority, event‐related potential, perceptual load, visual search

## Abstract

**Background:**

Attention selection is considered to be determined by the perceptual load of cognitive tasks and attention resources that are assigned to processing‐independent stimuli.

**Methods:**

Using visual search paradigm and Schulte grid, the behavioral responses and event‐related potential (ERP) of 27 pupils aged 8–11 (12 girls and 15 boys) were recorded. The subjects were asked to search for the numbers 1, 2, and 3 sequentially and locate the number 5 in the case of monochromatic (black) and bicolor (black‐red) numbers.

**Results:**

We found that the sequential search task took longer than the location search task (*p* < .05). Furthermore, both search tasks took longer in two‐color conditions than in monochromatic conditions. However, as for sequential search and location search tasks, no significant intra‐group difference was found. ERP data showed that there was significant difference between monochromatic and bichromatic conditions in locating search tasks (P2–P4: *p* < .05; T7: *p* < .05, T8: *p* < .05), but there was no significant difference in sequential search tasks.

**Conclusions:**

Our results indicates that red will be interfered when searching for one number, but will not be interfered when searching for three numbers, which is related to the higher perceptual load of sequential search tasks and less attention resources than those used to interfere with stimulus processing.

## Graphical abstract

The purpose of this study was to explore and compare the cognitive processes and brain electrical activity when red color appears in random spaces as either the target or interference during a visual search task among children. Our study is novel because we designed a dual‐color Schulte grid to simulate random events in life scenes in the visual search task.

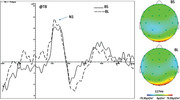

## 1 INTRODUCTION

Although the underlying mechanisms of attentive selection have attracted considerable attention over the past decade, there are still many controversies about how to control the processing of irrelevant information (Burnham, 2007). Both filter theory and attenuation theory assume that the brain's information processing capacity is limited (Zhu, 2000). Therefore, selective attention must adapt to the limitations of our cognitive capacity. However, it is not clear whether attentional selection runs in the early stage of information processing (Treisman & Gormican, 1988) or in the response phase (Duncan, 1980).The perceptual load theory which was proposed by Lavie et al. (2004) have made a further study on the theory of filter and attenuation. Lavie and Fox (2000) believed that attentional selection is determined by the perceptual load of the current task and irrelevant stimulus processing (Lavie & Cox, 1997). When the perceptual load of related tasks is high, there are fewer process resources for processing interfering stimuli. Therefore, interfering stimuli are passively excluded in the early stage of processing. However, when the perceptual load of the task is low, the perceptual ability is not consumed, not only in the early stages of processing, but also in later stages of processing, there are available resources to deal with interfering stimuli (Theeuwes, 1991; Yantis, 1993). In the experimental design, the perceptual load of tasks can be increased or decreased by changing the number of stimuli to be perceived (Eriksen & Eriksen, 1974) or adjusting the attention resources required for perceptual recognition of the same number of stimuli (Jonides & Yantis, 1988).

Color, especially red, is generally considered a single interference rather than a target task (Soto et al., 2005; Wolfe & Horowitz, 2004). The same space experiments have shown that in the process of visual attentional selection, the color of the stimulus takes precedence (Zhang et al., 2014). However, the experimental paradigm and specific tasks in the experiments often affect the ability of the color singularity to capture attention. Under the additional singular stimulus paradigm, color, as a singular stimulus can induce attentional capture (Theeuwes, 1991). Furthermore, Turatto and Galfano (2000, 2001) have used the target‐singular stimulus distance paradigm, and found that color singular stimuli can stimulate driven forms to capture attention. However, there are different results in irrelevant feature search paradigms. For example, Simons (2000), Chu and Zhou (2014) have both proved that a singular color stimulus does not significantly capture attention, because color acting as an interference and a target task simultaneously is a frequent occurrence in real life, and is related to spatial randomness.

The current research on attention selection mainly used eye movement trajectory and event‐related potentials (ERPs). Eye trajectory is a robust indicator of visual search processes, so the location of gaze point directly reflects the distribution of attention (Hu et al., 2012). The more fixation points, the longer the visual scanning path of eye movement, and the more resources required (Castelhano et al., 2008; Zhang et al., 2014).

Malcolm and Henderson (2009) divide the visual search process into start, search, and confirmation stages by using eye movement indicators. Eye movement experiments are usually performed using the image scanning paradigms, in which subjects are required to carefully examine the image (Borst et al., 2006; Finke et al., 1982; Liang & You, 2010). Luck (2009) has shown that visual stimulation induces the C1, P1, N1, P2, and other ERP components, of which P1 is sensitive to attention, related to the spatial direction of stimulus parameters (Hillyard et al., 1998) and also sensitive to subjects’ arousal (Vogel & Luck, 2000). In addition, Mangun (1995) has demonstrated that attention can significantly regulate the response of nerves to stimuli, as evidenced by high amplitudes of the N1 and P1 components in the primary visual cortex.

Recently, researchers have begun to pay attention to the plasticity of neural mechanisms of children's attention. For example, Sun et al. (2016) compared developmental patterns of attentional selection and inhibition between children (9–15 years old) and adults (19–29 years old), it is found that children showed greater plasticity in spatial attention than adults. In addition, Su and Gou (2007) monitored the visual and auditory pathways of children aged 5, 7, and 9 years and found that older children had the advantage of visual animation because they performed better when using flash or motion pictures than when using still pictures. Several training methods have been used to promote concentration, such as the teacher's learning methods, behavioral habits training, medical interventions, Schulte grid training, relaxation training, and spontaneous training (Xiao, 2016). The Schulte grid is a 5 × 5 grid composed of randomly distributed numbers or letters. The subjects are asked to focus on the center of the grid and use their peripheral vision (i.e., without moving their eyes.) to find all the numbers (or letters). The Schulte grid has been widely used in attention training for adults and children (Liu & Li, 2016; 2014; Yao, 2015).

To sum up, the interference effect of color on attention is usually based on experiments involving a single visual search task in the same space as the interference term. However, when the color is not only an interference item, but also a task target in a random space, does the interference effect of color still exist? Is there color interference when the target task involves multiple tasks?

In order to find answers to these questions, based on the image scanning experimental paradigm of Liang and You (2010), we designed a two‐color (black‐red) Schulte grid to simulate random events in life scenes. Black is a versatile and harmonious color (Zhang, 2016). It has been one of the most classic colors in China since ancient times and is often used in real life, so it is compared as a monochrome condition. The ERPs were recorded to measure electroencephalography (EEG) changes of children during visual search tasks (multitask: find numbers 1–3 in sequence; single task: find the number 5). Through this study, we explored the characteristics of EEG changes related to attention search, and whether color interfered with attentional selection (He et al., 2006a).

We assumed that (1) compared with the single visual search task of locating the number 5, the multitasking visual search of sequential search numbers 1, 2, and 3 will bring higher perceptual load in the case of a large number of perceptual tasks; (2) in the single‐task visual search, color (red) plays an interference role either as an interference item or as a target in random space. On the other hand, because the perceptual load of the multitask visual search paradigm is higher, too much attention resources cannot be allocated to deal with color interference, so color does not play an interference role in this kind of task.

## 2 MATERIALS AND METHODS

### 2.1 Subjects

A total of 27 fourth‐grade students (12 girls and 15 boys), aged 8 to 11 years old (mean, 9.8 years; standard deviation, 0.7 years), were recruited from a primary school in Shenzhen. The subjects had normal vision or correction, normal color discrimination, and were all right handed; none of the subjects had participated in similar experiments before; No subjects with ADHD were found. The written informed consents were obtained from all the students' parents or legal guardians.

#### 2.1.1 Visual experimental materials

**FIGURE 1:**
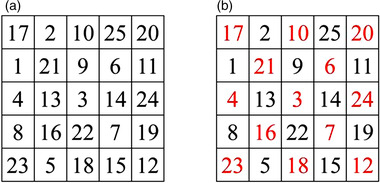
Pure black grid 001 and black‐red grid 001. (a) Subjects identified either digits 1, 2, and 3 in sequence or the digit 5 directly using the Schulte grid with pure black digits. (b) Subjects identified either digits 1, 2, and 3 in sequence or the digit 5 directly using the Schulte grid with black‐red digits

According to the Schulte grid (5 × 5) format, create 120 pure black digital pictures and 120 black‐red digital pictures, and then number them from 001 to 120. Each cell contains a random unique number between 1 and 25. The black‐red pictures of a number were created based on a picture of pure black number, and the number in the odd unit turns red. For example, a picture with the same number 001 (see Figure 1) shows the same numbers but different colors. Each picture is in JPG format with 1866 × 1866 pixels and a size of 158 KB. Using the E‐Prime 2.0 program, 120 pure black picture numbers and 120 black‐red digital pictures were compiled, and the pictures were displayed on the computer screen in a random order. The pictures used by each participant are random, so the pictures used are likely to be the same and possibly different. That was designed a dual‐color Schulte grid to simulate random events in life scenes.

### 2.2 Experimental design and procedures

The children were asked to identify the numbers 1, 2, and 3 in sequence or the number 5 directly using the Schulte grid. The time it took children to complete this task was recorded as the measure of their attention.

The experiment adopted the 2 × 2 within‐subject design with two factors: (a) search task: sequential search of numbers 1, 2, and 3 OR location search of number 5, and (b) color: pure black OR black‐red. The dependent variable was the time required to complete the search task, and the measurement indicators included the time taken to complete the task and the EEG components in the visual search process.

The experiment was carried out in a quiet ERP laboratory. The stimulus was displayed on a 23‐inch LCD screen (resolution: 1600 × 900, refresh rate: 60 Hz).The subjects sat at a distance of 75 cm from the computer display screen and looked directly at it during the test. The instructions were first displayed on the screen, and the experimental process and task requirements were explained to the subjects. The exercises were carried out after a full explanation to ensure that the subjects understood the task before starting the experiment. All the subjects participated in two trial runs to ensure the accuracy and smooth progress of the experiment.

**FIGURE 2:**
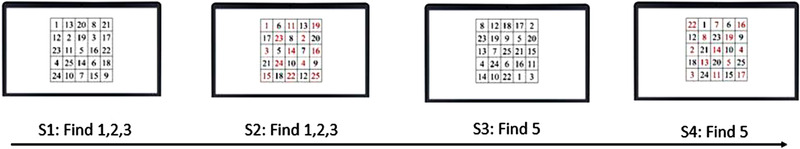
Experimental flow. S1 (pure black sequential search; BS):The subjects were asked to find numbers 1–3 in sequence and press the space bar upon finding number 3; S2 (black‐red sequential search; BRS): The subjects were asked to find numbers 1–3 in sequence and press the space bar upon finding number 3; S3 (pure black location search; BL): The subjects were asked to find the number 5 directly and press the space bar; and S4 (black‐red location search; BRL): The subjects were asked to find the number 5 directly and press the space bar

Participants were asked to press the spacebar to start a trial and press it again to move on to the next trial. The time between the two testes was recorded as the reaction time for each trial. After completing the program, subjects were allowed to have a full rest before moving the next program, until the following tests were completed (see Figure 2).

Each program randomly selected 27 test objects from 120 pictures through the E‐Prime program. Each subject tested only one picture in each program.

### 2.3 Data acquisition and processing

Data analysis was carried out on a Brain‐Product BP‐ERP workstation, and the 64‐channel electrode cap setting and the International EEG 10–20 system (American Electroencephalographic Society, 1994) were used. The Fcz electrode was used as a reference electrode for EEG recording and the geographic pole was positioned in the center of the forehead. EEG data were processed by the Brain Analyzer analysis software. Since the existing literature generally uses a1–a2 (BP's electrode cap tp9 is equivalent to A1 and TP10 is equivalent to A2) as the reference electrode, and as the electrode cap we used Fcz (reference electrode), the reference electrode will be replaced before analyzing the data in this study (Wu et al., 2017).

The band pass of the filter was 0.01–70 Hz, the sampling frequency was 1000 Hz, and the scalp resistance was less than 5 KΩ. The EEG from pre‐152 ms to post‐600 ms was analyzed, and the pre‐zero 200 ms was used as the baseline. Artifacts were automatically corrected, and those whose amplitudes were greater than ±100 μV were automatically eliminated in the stack. ERPs were obtained for each subject, in each experimental condition. Average ERP waveforms were computed based on the design of the experiment. We set up the measure windows based on the wave of the total average and previous studies (Zhen et al, 2011), and chose these outstanding wave peaks in occipital sites: (a) The N3, 210–400 ms of Fp1‐Fp2, Fz‐Fcz, Fc1‐Fc2 at the electrode sites in the frontal lobe were selected. (b) The N1, 80–200 ms and P2, 130–300 ms were the significant components of the P3‐P4 and P7‐P8 electrode sites in the parietal lobe and the T7‐T8 electrode sites in the temporal lobe. The peak and latency data of each waveform component were then analyzed to measure the attention resources required for the visual search task (He et al., 2006b; Lin et al., 2016; Wu et al., 2017; Zhen et al., 2011).

The statistical software package SPSS22.0 was used for statistical analysis. The peak and latency data of the anterior, middle, and posterior electrodes were all normally distributed and were expressed by mean ± standard deviation. Univariate analysis of variance (ANOVA) with post hoc tests and two‐way ANOVA were performed. The α value was set to 0.05. (He et al., 2006b; Lin et al., 2016).

## 3 RESULTS

### 3.1 Behavioral data analysis

The interaction between the color and the search method was analyzed (see Table 1) using two‐way ANOVA. We found that the search time of sequence numbers 1, 2, and 3 was higher than that of the location of number 5 in both groups. Further, the search times of the black‐red group were higher than of the pure black group under both conditions. Two‐way ANOVA revealed that in terms of search time, the subject effect of search task (F (1, 27) = 86.7, *p* < .0001) and color condition were both significant (F (1, 27) = 5.35, *p* = .023); we also found no significant interaction between the color condition and search methods (F (1, 27) = 0.18, *p* = .894).

**TABLE 1:** Behavioral data

Color condition (BR)	Search method (SL)		BR	SL	BR*SL
	Sequential search	Location search	F(*p*)	F(*p*)	F(*p*)
Pure black	4697 ± 1197	2445 ± 1005	5.35(0.000)	86.74(0.023)	0.02(0.894)
Black‐red	5217 ± 1345	3028 ± 1374			

The values are in ms; *n* = 27.

### 3.2 ERP data analysis

#### 3.2.1 Anterior part of the brain during the latent period and the peak

Table 2 shows the results of the statistical analysis of each electrode site N3 in the frontal lobes during the latent period and the peak. We found significant differences in the potential Fp2 (*p* = .042), Fc2 (*p* = .009), and Fcz (*p* = .014) sites under the black‐red sequential (BRS) and black‐red location search conditions (2, 4) during the latent period. However, there was no significant difference between pure black sequential search and pure black location search (1, 3), and also no significant difference between sequential search groups (1, 2) and location search groups (3, 4). No significant differences were observed in all the peak data.

**TABLE 2:** Statistical results for N3 in Fp2, Fc2, and Fcz sites during the latent period and the peak

		Pure black sequential search (1)	Black‐red sequential search (2)	Pure black location search (3)	Black‐red location search (4)	*p*
Fp2	Latent period (ms)	348 ± 63	317 ± 57	341 ± 54	351 ± 63	**.04** (2,4)
	Peak (μV)	−11.3 ± 6.6	−10.3 ± 9.1	−11.8 ± 5.9	−10.5 ± 5.9	**ns**
Fc2	Latent period (ms)	325 ± 60	293 ± 62	331 ± 56	339 ± 74	**.009** (2,4)
	Peak (μV)	−13.2 ± 7.6	−10.9 ± 8.0	−10.7 ± 5.7	−9.9 ± 5.1	**ns**
Fcz	Latent period (ms)	324 ± 58	296 ± 58	322 ± 53	337 ± 71	**.01** (2,4)
	Peak (μV)	−12.5 ± 6.8	−10.0 ± 7.4	−4.1 ± 43.5	−9.9 ± 5.0	**ns**

*n* = 27 in (1), (2), and (3); *n* = 26(remove one person's invalid data) in (4); values in bold indicate significant *p* values; ns, not significant against all other comparisons.

**FIGURE 3:**
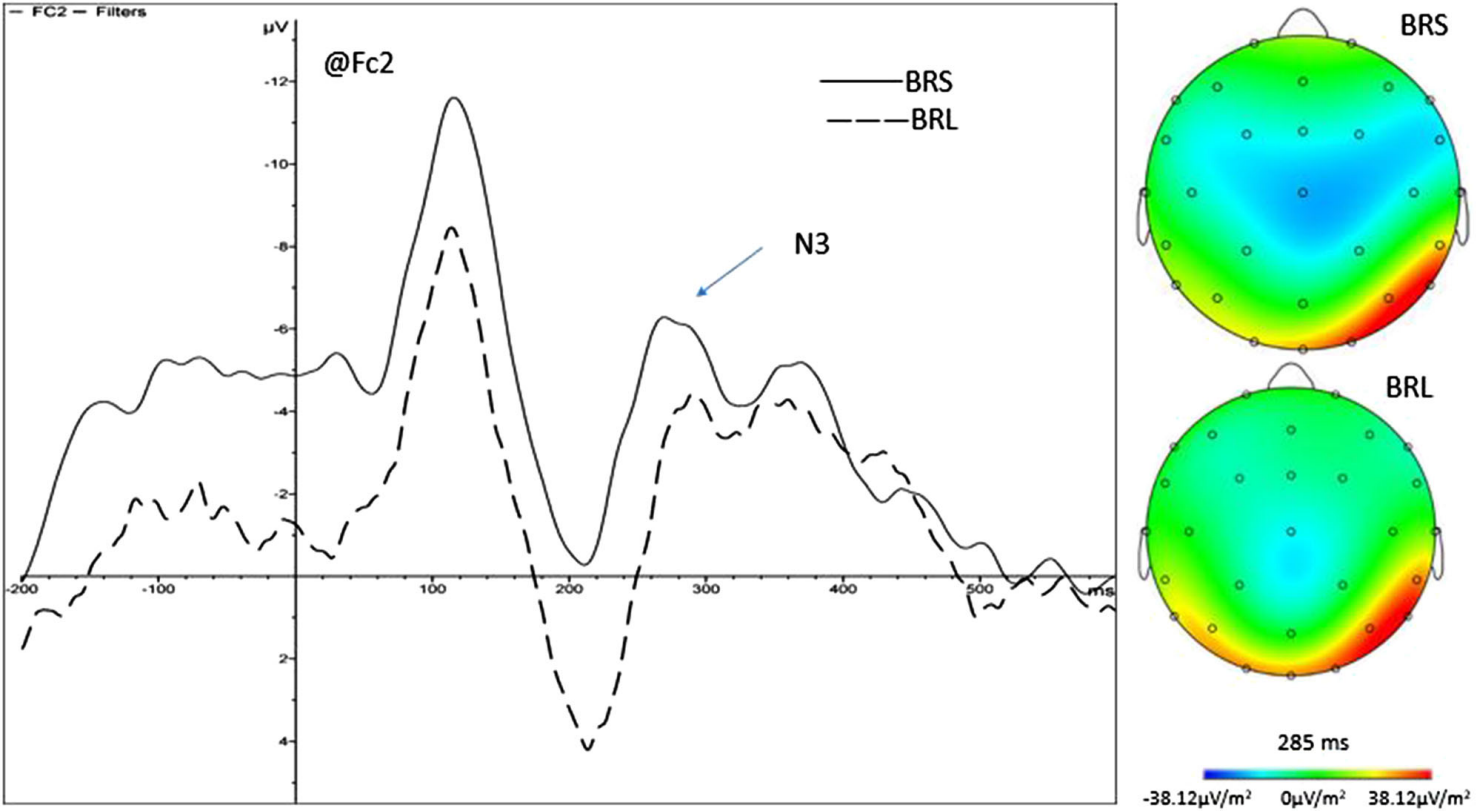
ERP waveforms and corresponding EEG activation patterns at the Fc2 site in the black‐red group. Comparison of ERP waveforms and EEG activations at the Fc2 site in the parietal lobe and the peak during the pure black‐red sequential search (BRS) and black‐red location search (BRL) conditions. ERP, event‐related potential; EEG, electroencephalograph

Figure 3 shows the ERP wave forms and the corresponding EEG activation patterns of the Fc2 site in the black‐red group. The ERP waveform makes us intuitively see the significant difference in the position of Fc2 in N3 under the condition of the pure BRS search and BRL search; this significant difference can also be seen in EEG activation patterns.

#### 3.2.2 Mid‐brain during the latent period and the peak

Tables 3 and 4 summarize the results of statistical analysis of N1 and P2 at each electrode site in the mid‐brain during the latent period and the peak, respectively. We found a significant difference between the pure black sequential search and pure black location search conditions (1, 3) in the T8 (*p* = .05 = .05) site (see **N1,**Table 3) during the latent period. We also found a significant difference between the pure black location search and BRL search conditions (3, 4) in the potential P4 (*p* = .034 < .05), T7 (*p* = .033 < .05), and T8 (*p* = .030 < .05) sites (see **P2,** Table 4). However, there was no significant difference among the following groups: sequential search groups (1, 2), pure black sequential search and BRL search (1, 4), black‐red search groups (2, 4). There were no significant differences in all the peak data.

**TABLE 3:** Statistical results for N1 in the T8 site within the mid‐brain during the latent period

		Pure black sequential search (1)	Black‐red sequential search (2)	Pure black location search (3)	Black‐red location search (4)	Significance (post hoc pairwise comparison)
T8	Latent period (ms)	95 ± 22	99 ± 18	103 ± 17	100 ± 15	***p* = .050** (1,3)
	Peak (μV)	−6.7 ± 6.	−7.6 ± 5.2	–7.0 ± 5.0	–7.3 ± 7.3	**ns**

*n* = 27 in (1), (2), (3), and (4); values in bold indicate significant *p* values; ns, not significant against all other comparisons.

**TABLE 4:** Statistical results within the mid‐brain during the latent period and the peak

		Pure black sequential search (1)	Black‐red sequential search (2)	Pure black location search (3)	Black‐red location search (4)	Significance (post hoc pairwise comparison)
P4	Latent period (ms)	239 ± 44	243 ± 39	234 ± 34	257 ± 42	***p* = .034** (3,4)
	Peak (μV)	−6.9 ± 5.1	−7.6 ± 5.2	−6.9 ± 4.9	−7.3 ± 4.7	**ns**
T7	Latent period (ms)	228 ± 23	237 ± 29	222 ± 22	238 ± 27	***p* = .033** (3,4)
	Peak (μV)	17.3 ± 9.8	18.6 ± 11.1	16.5 ± 11.2	15.6 ± 11.0	**ns**
T8	Latent period (ms)	240 ± 33	253 ± 39	240 ± 26	260 ± 35	***p* = .030** (3,4)
	Peak (μV)	5.3 ± 4.6	5.9 ± 5.0	6.3 ± 4.8	5.4 ± 4.7	**ns**

*n* = 27 in (1), (2), and (3); *n* = 26 in (4); values in bold indicate significant p values; ns, not significant against all other comparisons.

**FIGURE 4:**
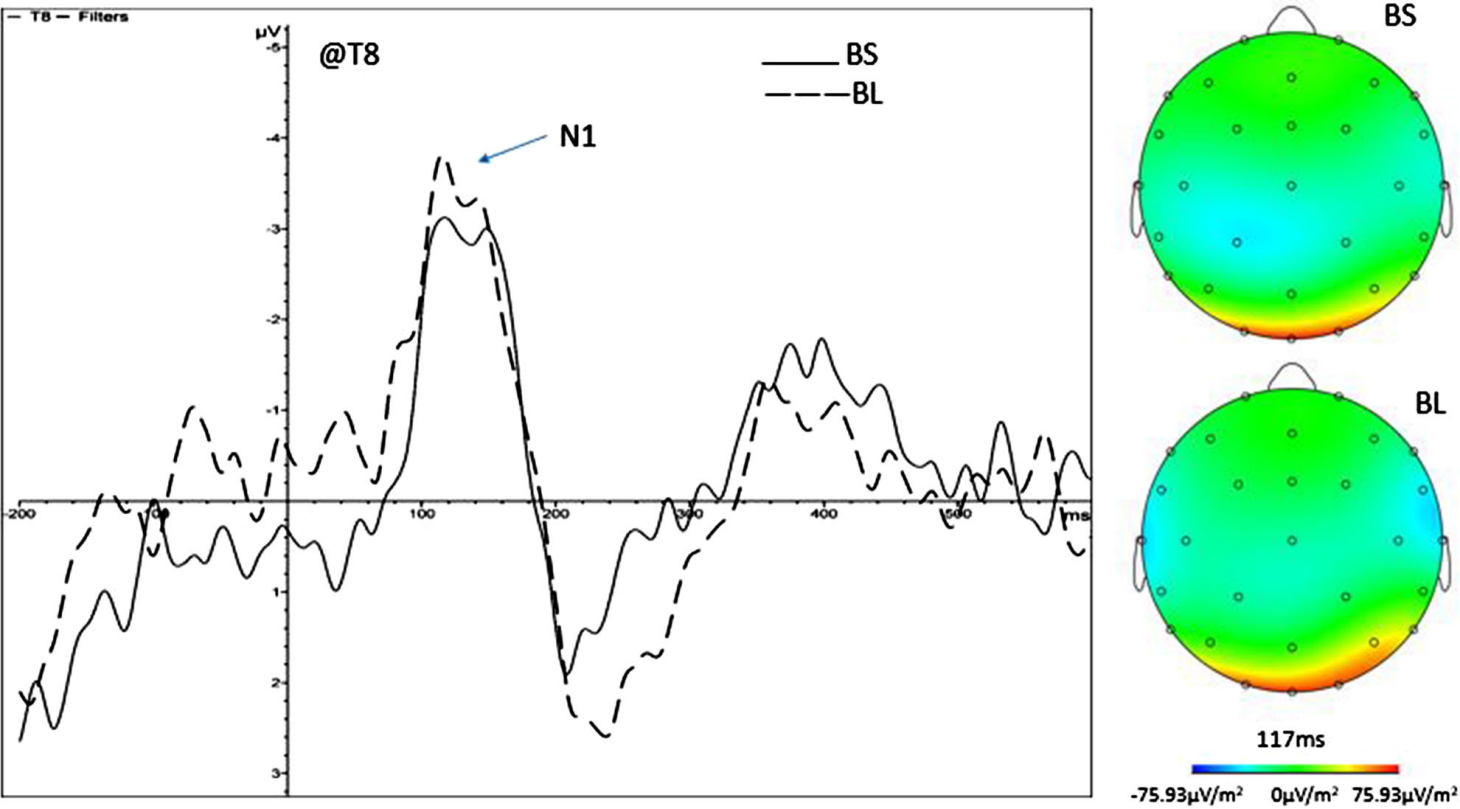
ERP waveforms and EEG activation patterns at the T8 site in the pure black group. Comparison of ERP waveforms and EEG activations at T8 of the parietal lobe and the peak during the pure black sequential search (BS) and pure black location search (BL) conditions. ERP, event‐related potential; EEG, electroencephalograph

In Figure 4, the ERP waveform and EEG activation patterns of T8 in the pure black group were compared, and it was found that there was a significant difference between the pure black sequential search and the pure black location search (1, 3) in N1 position. The pattern of EEG activation showed a significant difference between the pure black sequential search and the pure black location search, because the area of activation in the middle of the brain is different between the two.

**FIGURE 5:**
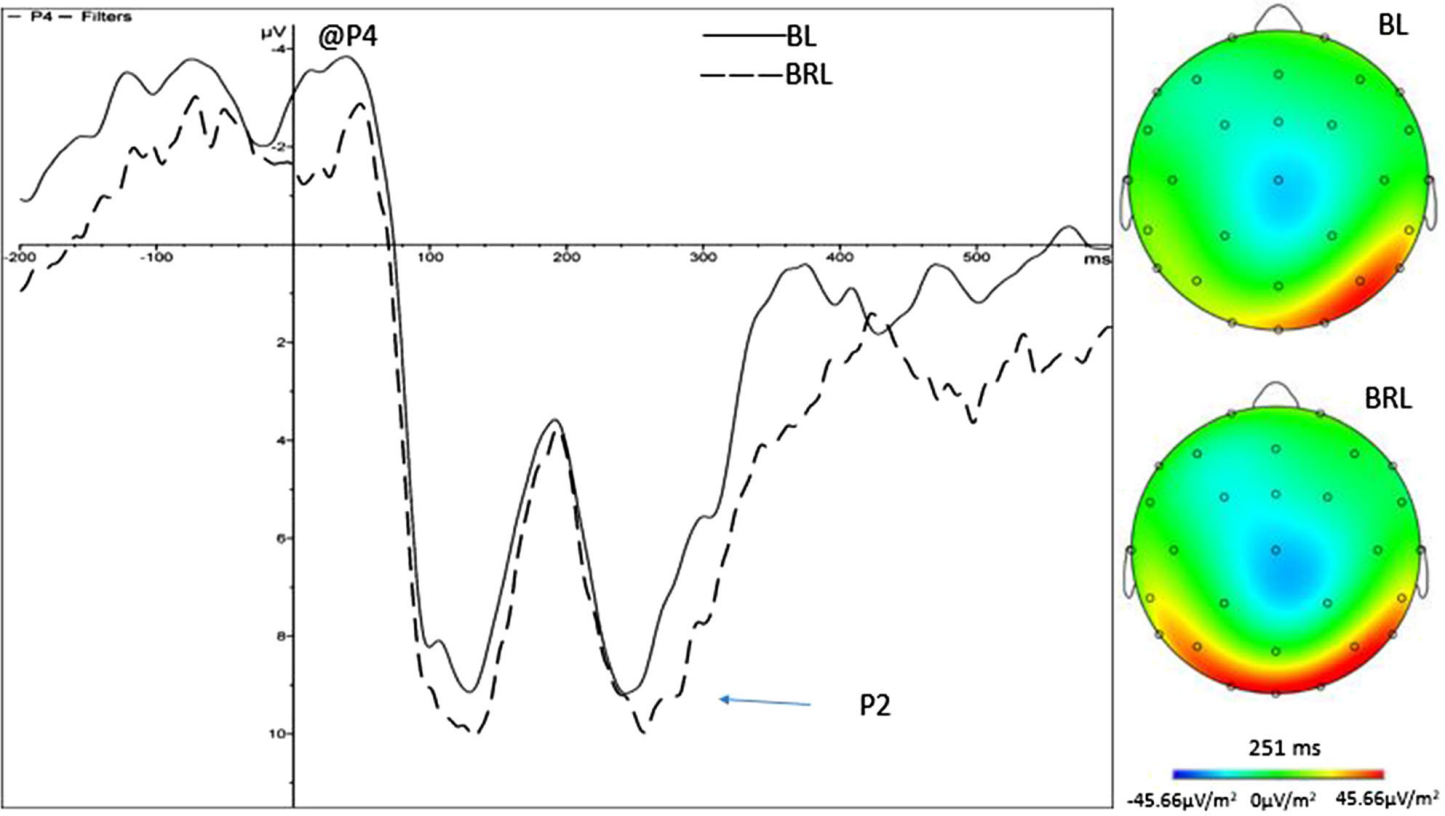
ERP waveforms and EEG activation patterns at the P4 site in the location search groups. Comparison of ERP waveforms and EEG activation at P4 of the parietal lobe and the peak during the pure black location search (BL) and black‐red location search (BRL) conditions. ERP, event‐related potential; EEG, electroencephalograph

In Figure 5, the location search group in P4 is compared by ERP waveform and EEG activation mode. ERP waveform showed significant difference in the position of P2 between pure black location search and BRL search. EEG activation patterns showed a significant difference in the activation state of the mid‐brain during 251 ms (P2) between pure black location search and BRL search.

## 4 DISCUSSION

### 4.1 Perceptual load of search tasks

We found that the search time for locating numbers 1, 2, and 3 in a sequence was longer than that for locating the number 5 through location search under the monochrome and bichrome conditions. We found that there were significant differences between the BS (pure black sequential search) and BL (pure black location search) search conditions, and between the BRS and BRL search conditions. This may be because searching for three numbers requires more resources and a higher perceptual load than searching for one number. The higher perceptual load was caused by the increase in the number of items to be focused on as part of the experiment (Eriksen & Eriksen, 1974). Our ERP findings suggest that in the pure black group, the latency of the N1 component at T8 was significantly different between the sequential search and location search conditions. Similarly, in the black‐red group, the latencies of Fp1, Fc1, and Fcz in the N3 component were significantly different between the sequential search and location search conditions. Based on these observations, it was inferred that (1) there was a difference in the perceptual load between the sequential search for numbers1–3 and location search for number 5; and (2) the search time for locating numbers1‐3 was longer than for searching number 5, which indicated that the search for three numbers was a high perceptual load, and the search order number 5 was a low perceptual load. Our observations support the previous theory—increasing the perceptual load increases the number of perception in the experimental design.

### 4.2 Color‐related interference

Brain regions, associated with visual attention processing, are the dorsolateral prefrontal cortex, the subcutaneous cortex, and the temporal epithelium (Peng, 2012). These brain regions control the cognitive processes that regulate attention. In this study, we found that there were significant task‐related differences in the ERPs at sites Fp2, Fc2, and Fcz in the frontal lobe, P4 in the parietal lobe, and T7 and T8 in the temporal lobe. This suggests that the frontal, parietal, and temporal lobes were all active in this experiment and might have been involved in the search task. But why significant task‐related differences in the ERPs were found symmetrically in the temporal lobe (T7 & T8) but asymmetrically in the frontal lobe (only Fp2, Fc2 but not Fp1, Fc1) and parietal lobe (P4 but not P3)? The parietal and frontal lobes may have a unilateral advantage in numerical processing (Arsalidou et al., 2010), but it is not obvious in the temporal lobe (Zhang et al., 2005).

Under the conditions of BL and BRL search, there were significant differences in latency of P4 (right parietal), T7 (left middle temporal), and T8 (right middle temporal).Red is thought to be a significant color that can attract our attention, and the study also supported this observation. In the location search task, the P2 components, related to the pure black and black‐red search conditions were significantly different in the right parietal and bilateral temporal lobes, indicating that red color may be preferred in the attentional capture process; so the BRL search task took more time than the BL search task. Therefore, the red color might have an interference effect on the completion of the location search task. Our experimental results are consistent with previous studies (Turatto & Galfano, 2000; 2001; Yantis, 1993).

The resource allocation theory holds that attention has a characteristic called finiteness, which is attributed to the limitation of psychological operation resources, and that the main mechanism attention is resource allocation (Kahneman, 1973). We found differences in the allocation of attentional resources between the sequential search of numbers 1, 2, and 3 and location search of number 5, and the former allocated more attention than the latter. Therefore, the time to complete the sequential search was significantly different from the time to location search tasks.

Interestingly, we found that there was no significant difference in the brain response times related to the BS and BRS conditions. On the contrary, we found significant differences in the brain response times associated with the BL and BRL conditions (at P4, T7, and T8 in P2), which can be explained by the perceptual load theory (Lavie & Tsal, 1994). The sequential search task involved searching for numbers 1, 2, and 3, while the location search task involved searching for only the number 5. The perceptual load of the sequential search was relatively higher, which consumed the limited attentional resources. Therefore, the interference stimulus, the red color, which had nothing to do with the target, was not perceptively processed, so there was no interference effect. In contrast, the perceptual load of the location search task was relatively lower, and only a part of the attention resources was invested in the process. As a result, the excess attention resources and the interference stimulus (red color), will be processed, resulting in interference effects (Lavie et al., 2004).

This study addressed the primary school students’ patience and limited energy and other factors. Further, as participants produce only a single response, it is difficult to predict what participants were actually doing during the task and whether they followed exact instructions. Future studies should focus on adults and more detailed behavioral responses studies need to be designed.

## 5 CONCLUSION

In summary, we found that when a cognitive task involves locating a single target, the color (red) is captured and processed as an interference signal in the random space of the Schulte grid. However, under the same conditions, when the task involves locating multiple (three) targets, due to the high perceptual load, there is no interference effect related to the color stimulus, and no additional attentional resources are available for processing the color stimulus.

## CONFLICT OF INTEREST

The authors declare no conflict of interest.

## AUTHOR CONTRIBUTIONS

Aiqiang Lu and Qiuyi Zhongcheng designed this study. Qiuyi Zhongcheng collected the data. Shengxi He and Aiqiang Lu analyzed the data. Aiqiang Lu wrote the manuscript. Dongmei Wang, Wei Zhang, Zezhi Li, and Shengxi He read and approved the final manuscript.

### PEER REVIEW

The peer review history for this article is available at https://publons.com/publon/10.1002/brb3.2471

## Data Availability

The data used to support the findings of this study are available from the corresponding author upon request.
